# Tryptophan Research in Panic Disorder

**DOI:** 10.4137/ijtr.s929

**Published:** 2008-08-14

**Authors:** Eduard Maron, Jakov Shlik, David J. Nutt

**Affiliations:** 1 Research Department of Mental Health, The North Estonian Regional Hospital, Psychiatry Clinic, Tallinn, Estonia; 2 Department of Psychiatry, University of Tartu, Tartu, Estonia; 3 Estonian Genome Project, University of Tartu, Estonia; 4 Department of Psychiatry, University of Ottawa, Ottawa, Ontario, Canada; 5 Department of Community Based Medicine, Psychopharmacology Unit, University of Bristol, U.K

**Keywords:** tryptophan, panic disorder, anxiety, challenge, gene

## Abstract

A considerable body of evidence suggests the involvement of serotonin neurotransmission in the pathogenesis of panic disorder. Research on pathways and functions of tryptophan, an essential amino acid converted into serotonin, may advance our understanding of serotonergic actions in panic disorder and related phenomena. The investigative approaches in this field include manipulations of tryptophan availability as well as genetic association and functional brain imaging studies. In this review we examine the principle findings of these studies and propose further research directions.

Panic disorder (PD) is a prevalent and serious illness in need of a better understanding of its neurobiological basis to improve treatment outcomes. The pathogenesis, clinical manifestations and treatment effects of PD are thought to be substantially related to the functions of serotonin or 5-hydroxytryptamine (5-HT) neurotransmission. The crucial role of 5-HT in PD has been suggested by clinical studies demonstrating that medications specifically increasing the synaptic availability of 5-HT, especially the selective 5-HT re-uptake inhibitors (SSRIs), are particularly effective in the treatment of PD (Nutt, 1998). Extensive experience with SSRIs in the treatment of PD and the effect of tryptophan depletion (TD) to undermine this action underscored the necessity of increased synaptic availability of 5-HT for achieving remission. Furthermore, growing data from experimental and neuroimaging studies have suggested that altered availability of brain 5-HT is associated with PD; however the exact mechanisms of a possible disturbance in 5-HT metabolism are not fully understood (Maron and Shlik, 2006). 5-HT is synthesized from the essential amino acid tryptophan via intermediate metabolite, 5-hydroxytryptophan (5-HTP), and stored in reserpine-sensitive vesicles until released into the synaptic cleft by nerve impulses. The conversion of tryptophan into 5-HTP is regulated by tryptophan hydroxylase (TPH), the rate limiting enzyme in biosynthesis of 5-HT. In the next step, 5-HTP is decarboxylated by aromatic acid decarboxylase to 5-HT. Unlike 5-HTP, tryptophan can be shunted into kynurenine via tryptophan 2,3-dioxygenase, making tryptophan unavailable for 5-HT production (Birdsall, 1998; Ruddick et al. 2006). TPH can be inhibited by numerous factors, including stress, insulin resistance, vitamin B6 deficiency, and insufficient magnesium, that may increase the conversion of tryptophan to kynurenine. Under normal conditions, the enzyme TPH is only about 50% saturated in brain, therefore the synthesis of 5-HT is dependent on the availability of free plasma tryptophan, whereas alterations in its availability correlate with the amount of synthesized 5-HT (Schaechter and Wurtman, 1990). Presently, the central 5-HT pathways remain the main targets in the research on the neurobiology of PD. In this paper we review the studies focusing on the role of tryptophan and discuss research directions in this area.

## Experimental Studies

Deakin and Graeff (1991) have proposed that in certain brain regions 5-HT restrains panic behaviour and therefore 5-HT deficit may predispose to panic reactions. Two complementary approaches in altering the tryptophan availability were found helpful to understand the role of 5-HT in the pathophysiology of PD. First is the acute tryptophan depletion (ATD) achieved by a dietary manipulation leading to a rapid reduction in plasma tryptophan levels, typically 70%–80% reduction within 5 hours (Young et al. 1985). The drop in the peripheral source of 5-HT is reflected by a substantial decrease in the brain 5-HT levels. For instance, the rate of 5-HT synthesis in vivo using positron emission tomography (PET) was reduced up to 40% of baseline values after ATD in healthy subjects (Nishizawa et al. 1997). The methods and psychopharmacological rationale of ATD as well as its application in psychiatric research have been recently reviewed in detail (Hood et al. 2005; Bell et al. 2005). An opposite effect of tryptophan supplementation could be achieved with the use of 5-HT precursors, particularly 5-HTP, which increases brain concentrations of 5-HT (den Boer and Westenberg, 1990; van Vliet et al. 1996). Both methods could be more salient under conditions of experimental challenge with panicogenic agents, such as carbon dioxide (CO2), cholecystokinin-tetrapeptide (CCK-4), a CCK brain subtype receptor agonist, or benzodiazepine receptor antagonist, flumazenil. The main results from the studies investigating the effects of 5-HT synthesis rate manipulation on panic responses are summarized in Table 1.

### Depletion studies

ATD alone is not anxio- or panicogenic in unmedicated PD patients or healthy subjects (Goddard et al. 1994, 1995; Klaassen et al. 1998) but may increase the susceptibility to panic under challenge conditions. ATD was associated with an increased respiration and subjective breathlessness in patients with PD as compared to controls in response to 5% CO2 challenge, but no significant between-group differences were observed on primary measures of panic or anxiety (Kent et al. 1996). Klaassen et al. (1998) have shown that in healthy subjects ATD significantly increased 35% CO2-induced somatic, but not cognitive, panic symptoms in male subjects with a similar panic rate in ATD and placebo groups. Koszycki et al. (1996) have found that ATD did not influence the panicogenic effect of CCK-4 in healthy males, although it did augment CCK-4-mediated neuroendocrine activation. Further studies have mostly proven that ATD increases the sensitivity to CO2 challenge in patients with PD. Specifically, Miller et al. (2000) showed that ATD caused a greater panic and anxiogenic response and a higher rate of panic attacks after 5% CO2 inhalation in PD patients, but not in healthy subjects. Another study in patients with PD by Schruers et al. (2000) demonstrated a significant increase in anxiety and panic symptoms induced by 35% CO2 inhalation in the ATD group when compared to the placebo condition. Recently, Hood et al. (2006) assessed the effects of ATD on neuroendocrine, autonomic and psychological responses to single breath 35% CO2 inhalation in healthy volunteers. They observed no exacerbation of the provoked anxiety symptoms and no increase in the psychological response to a challenge stressor.

A study by Bristol group found that ATD reversed the antipanic effect of chronic treatment with the SSRI paroxetine in PD patients, manifested as an increase in panicogenic response to a challenge with flumazenil (Bell et al. 2002). This finding supported the hypothesis that a decrease in 5-HT neurotransmission predisposes to panic attacks and that the antipanic effect of SSRIs depends upon the availability of 5-HT in the brain (Nutt et al. 1999). Later, the same group demonstrated that ATD also counteracted the therapeutic effect of SSRIs in patients with social anxiety disorder (SAD). Particularly, they found that ATD induced a significant increase in anxiety when listening to an anxiogenic autobiographical script, supporting the idea that 5-HT is important in maintaining the response to SSRIs in SAD (Argyropoulos et al. 2004). In the next study they aimed to characterize the effect of ATD on cardiovascular and psychological responses to stress challenge in recovered patients with PD or SAD (Davies et al. 2006). The results showed that both systolic and diastolic blood pressure responses to stress were significantly greater under ATD than control condition, as were the psychological or anxiety responses to stress, suggesting that 5-HT is involved in the control of both cardiovascular and psychological aspects of acute stress response. The lack of correlation between blood pressure and psychological responses to stress in this study indicated that 5-HT may affect cardiovascular and psychological domains independently rather than the cardiovascular responses being secondary to psychological changes.

Recently, Tartu-Bristol collaborative study has extended this line of research by evaluating the effect of ATD on CCK-4-induced symptoms in patients with PD who had responded to the treatment with a SSRI citalopram (Tõru et al. 2006). The results of this study showed that ATD did not affect behavioural or cardiovascular response to a CCK-4 challenge in the responders after 10-week treatment with citalopram, demonstrating that an acute decline in the central availability of 5-HT does not reverse the protective effect of SSRI treatment on CCK-4-induced panic. This finding suggested that the reduced sensitivity to CCK-4 after SSRI treatment may be due to mechanisms other than an increase in 5-HT availability in the brain, and that the panicogenic effects of CCK-4 and flumazenil may be modulated differently by 5-HT. Interestingly, the studies examining effects of ATD in obsessive-compulsive disorder (OCD) in patients remitted on SSRI, either on medication or drug-free, failed to show any significant exacerbation in OCD or related symptoms under ATD condition at rest (Barr et al. 1994; Smeraldi et al. 1996; Huwig-Poppe et al. 1999; Külz et al. 2007) or during symptom provocation (Berney et al. 2006). The lack of effect of ATD on OCD symptoms suggests that the durability of the effects of SSRI in OCD may be less dependent on the synaptic availability of 5-HT.

### Supplementation studies

Early studies with direct administration of tryptophan did not demonstrate any differences in the reactivity between patients with PD and healthy subjects. Specifically, Charney et al. (1982) showed that intravenous infusion of L-tryptophan was not accompanied by anxiety symptoms, although it induced significant increases in prolactin and growth hormone in healthy subjects as compared to placebo injection. Later they also established that the ability of tryptophan to increase prolactin levels was not different between the PD patients and healthy subjects and was not influenced by alprazolam treatment (Charney and Heninger, 1986). Notably, a clinical observation was made that oral tryptophan augmentation did not potentiate the beneficial effect of 10-week treatment with clomipramine on phobic avoidance, phobic fears or the incidence of panic attacks in a small sample of patients with agoraphobia or social phobia (Pecknold et al. 1982).

Studies with 5-HTP have shown that administration of this immediate precursor of 5-HT had beneficial effects on panic attacks in patients with anxiety disorders (Kahn and Westenberg, 1985; Kahn et al. 1987). Also it was reported that an acute administration of 60 mg of 5-HTP was not anxiogenic in healthy subjects and was felt as a relief in patients with PD, whereas 5-HTP infusion led to substantial, but similar increases in plasma cortisol, beta-endorphin and melatonin levels in both groups (den Boer and Westenberg, 1990). Later, van Vliet et al. (1996) assessed the psychological and hormonal effects of 5-HTP in small samples of PD patients and healthy controls who received an i.v. challenge with 10, 20 and 40 mg of 5-HTP and placebo in random order on four different occasions. No panic or anxiety symptoms were observed in any of the groups during and after infusion of placebo or different doses of 5-HTP. Only infusion with 40 mg 5-HTP led to an increase in plasma cortisol in both patients and controls, whereas rise in plasma cortisol level was higher in PD patients only 30 min after infusion. Conversely, Schruers et al. (2002b) have found that the administration of 200 mg 5-HTP restrained panic responses to 35% CO2 challenge in patients with PD, but not in healthy subjects. Furthermore they detected a significant rise in salivary cortisol levels in both patients and controls following acute administration of 5-HTP, whereas no such effects were seen after placebo (Schruers et al. 2002a). Our study in healthy volunteers has shown that 200 mg of 5-HTP significantly lowered the panic rate and intensity of cognitive symptoms of panic in females and the intensity of somatic symptoms of panic in males (Maron et al. 2004b). These findings suggested that an increased availability of 5-HT may have a gender-dependent protective effect in CCK-4-induced panic. We also observed a significantly greater increase in blood pressure after CCK-4 injection in 5-HTP-group in comparison to placebo with a similar trend in heart rate. Although it is known that 5-HT has vasotonic properties, the mechanism of these paradoxical cardiovascular effects of 5-HTP during CCK-4-induced panic is not clear.

In summary, most of the above-mentioned studies demonstrate that a decrease in 5-HT synaptic availability by ATD increases susceptibility to panic and, conversely, an increase in 5-HT neuro-transmission particularly by administration of 5-HTP has antipanic effects. The lack of such effects with L-tryptophan could probably be explained by competing pathways of the synthesis of 5-HT and kynurenine. Notably, the use of higher doses of 5-HTP demonstrated more prominent anti-panic effects. Although it is quite clear that acute pre-treatment with 5-HTP increases the net amount of released 5-HT and its synaptic availability (Dreshfield-Ahmad et al. 2000; Fickbohm and Katz, 2000), the possible antipanic action mode of 5-HTP requires further scrutiny. The increase in plasma cortisol and prolactin following 5-HTP administration is modulated by different postsynaptic receptors, including 5-HT1A, 5-HT2A, 5-HT2C, and 5-HT3 (Meltzer and Maes, 1994; Meltzer et al. 1997). Pre-treatment with pindolol, a 5-HT1A partial antagonist, significantly inhibited the prolactin, but not the cortisol response to 5-HTP (Meltzer and Maes, 1994), whereas ritanserin, a 5-HT2A/5-HT2C antagonist, did not block the prolactin response to tryptophan (Deakin, 1996) and even increased it in another study (Charig et al. 1986). It is not known whether neuroendocrine and antipanic effects of 5-HTP are mediated via the same 5-HT receptors. Therefore, further experimental studies with selective 5-HT-ergic antagonists are needed to clarify the antipanic action of 5-HTP and its clinical relevance. A fundamental question to be addressed in further studies is whether the supposed 5-HT deficit is a primary factor in the manifestations of PD or 5-HT enhancement exerts a protective influence through projections on other neurotransmission systems, which are responsible for triggering panic attacks.

## Genetic Studies

There have been increasing efforts to determine genes in 5-HT system related to vulnerability to PD. TPH gene has emerged as a major candidate for genetic association studies in many psychiatric disorders, including PD. Recently, the two genes coding TPH have been differentiated as TPH1 and TPH2 (Walther et al. 2003). The association studies with TPH1 gene have shown lack of association between its polymorphisms, such as 1095T/C, 218A/C or 779A/C and PD (Fehr et al. 2001; Han et al. 1999; Maron et al. 2005a, b). Zill et al. (2004b) have established that human TPH1 and TPH2 are expressed in nearly equal amounts in several brain regions such as the frontal cortex, thalamus, hippocampus, hypothalamus and amygdala, while TPH2 is predominant in the brain stem, the major locus of the 5-HT-producing neurons, and TPH1 mRNA is exclusively present in peripheral tissues. A number of studies have now indicated the associations between several TPH2 polymorphisms and various psychiatric disorders (Zhang et al. 2004). Possible associations were found between various TPH2 polymorphisms and major depression (Zill et al. 2004a; Zhang et al. 2005; Zhou et al. 2005), response to antidepressants (Peters et al. 2004), suicidal behaviour (Zill et al. 2004c; Zhou et al. 2005), ADHD (Sheehan et al. 2005; Walitza et al. 2005), autism (Coon et al. 2005), bipolar disorder (Harvey et al. 2004) and OCD (Mössner et al. 2006b). In contrast, other studies failed to find associations between TPH2 polymorphisms and suicidality or mood disorders (De Luca et al. 2004, 2005; Garriock et al. 2005). Despite the fact that TPH2 appears to be principally responsible for the synthesis of 5-HT in the brain (Zhang et al. 2004), the functional meaning of TPH2 polymorphisms as well as their regulatory effect on the 5-HT synthesis rate remain unclear (Breidenthal et al. 2004).

Presumably, TPH2 rather than TPH1 gene polymorphisms are more relevant targets in PD due to a stronger relevance of TPH2 to the regulation of 5-HT neurotransmission. To test this hypothesis we compared the distributions of TPH2 gene polymorphisms in the samples of Estonian patients with PD (n = 213) and matched healthy subjects (n = 303) (Maron et al. 2007). Two polymorphic loci from the TPH2 gene were included in our study: rs1386494, previously associated with major depression by Zill et al. (2004a) and rs1386483, recently associated with impulsivity (Stoltenberg et al. 2006). Overall we found no significant associations between these SNPs and PD. These findings were similar to the results of the by Mössner et al. (2006a), where no associations were seen between PD and two other common SNPs, rs4570625 and rs4565946, located respectively in the putative transcriptional control region and in the intron 2 of TPH2. Thus, data so far seem to disprove the hypothesis of a link between genetic variations in the TPH2 gene and susceptibility to PD. Nevertheless, our separate analyses by gender revealed a positive association with rs1386494 SNP in females with pure PD phenotype (n = 52), but not in the total female group (n = 163) or in the subgroup of females with PD and affective comorbidity, including major depression (n = 111). Particularly, we detected that G/G genotype and G allele were less frequent in females with pure PD as compared to control females (p = 0.01 and p = 0.02, respectively), indicating a possible gender-specific effect of rs1386494 variants in pure phenotype of PD (Maron et al. 2007) In order to further clarify the involvement of this SNP in PD, we recently examined its effect on the vulnerability to CCK-4-induced panic attacks in 47 male and 63 female healthy subjects (Tõru et al. unpublished). We detected significant associations between rs1386494 and the rate of panic attacks induced by CCK-4 injection. Particularly, both G/G genotype and G allele were more frequent in panickers as compared to non-panickers (p = 0.03 and p = 0.009, respectively). Further analyses showed that these associations remained significant only in female subjects. Thus, the challenge test confirmed the involvement of TPH2 genetic polymorphism, rs1386494, in panicogenesis and particularly in susceptibility to panic attacks. However, the opposite direction of association between the patients and healthy subjects complicates the interpretation of these findings.

## Brain Imaging Studies

The visualization of neurochemical processes has provided more evidence for the involvement of 5-HT-ergic pathways in panic circuitry. Using the SPECT tracer [^123^I]nor-β-CIT we demonstrated that symptomatic patients with PD had significantly lower 5-HT transporter (5-HTT) binding in the midbrain raphe, in the temporal lobes, and in the thalamus than the healthy controls. The patients with PD in remission had normal 5-HTT-binding properties in the midbrain and in the temporal regions, but still a significantly lower thalamic 5-HTT binding (Maron et al. 2004a). Furthermore, two positron emission tomography (PET) studies have revealed a marked reduction of 5-HT1A receptor binding in many brain regions in PD using the tracer WAY100635. Particularly, PD patients showed significantly decreased 5-HT1A receptor binding in the anterior and posterior cingulate cortices, and in the midbrain raphe in comparison to healthy controls without any between-group differences in the anterior insula, the mesiotemporal cortex, and the anterior temporal cortex (Neumeister et al. 2004). Another PET study has demonstrated a reduced binding to 5-HT1A receptors in the raphe region as well as in the amygdala, and the orbitofrontal and temporal cortices in untreated patients with PD (Nash et al. in press). PD patients who fully recovered after treatment with SSRIs in this study showed normalized density of postsynaptic receptors, but there remained a reduction in the density of 5-HT1A receptors in the raphe and in the hippocampus, suggesting a trait nature of these alterations. However no significant difference was seen in global postsynaptic binding for treated patients versus controls. Based on these data and the hypothesis of 5-HT deficit in PD, we suggested that such alterations in central 5-HT system in PD could reflect a compensatory process attempting to increase 5-HT neurotransmission to inhibit the panic (Maron and Shlik, 2006).

To prove this assumption a measurement of 5-HT synthesis rate in the brain of patients with PD would be warranted. Of interest, a decreased index of 5-HT synthesis, as measured by PET with α-[11C]methyl-l-tryptophan trapping, was detected in parts of the limbic and paralimbic cortices of patients with major depression (Rosa-Neto et al. 2004). Another PET study demonstrated a significant increase in 5-HT synthesis rate in the prefrontal cortex of patients with depression who had received treatment with SSRI citalopram alone or with pindolol (Berney et al. 2008). Considering that major depression is the most often comorbid condition in patients with PD, and that principal therapeutic mechanism of SSRIs could be similar for both conditions, further PET studies of 5-HT synthesis rate in PD should be of interest.

## Conclusive Remarks

Tryptophan-related research has significantly extended our knowledge on the role of 5-HT in PD. Although a number of studies manipulating tryptophan availability provide reasonable evidence for a protective influence of 5-HT against panic responses, some of the findings are divisive, indicating that the role of 5-HT in PD is not unique and 5-HT may interact differently with relevant neurotransmitter systems. Recent genetic studies tentatively point to the function of TPH2 gene in a predisposition to panic, but these data need to be replicated and extended. The functional role of TPH2 genetic polymorphisms on tryptophan metabolism requires further elucidation. The characterisation of molecular mechanisms underlying tryptophan metabolism in different neuronal populations might lead to novel therapeutic strategies based on specific regulation of different metabolic pathways for tryptophan (Ruddick et al. 2006). Such studies may also shed more light on the relatioships between pathways of tryptophan metabolism and features of PD in it’s different clinical stages. The gender difference in the role of 5-HT in panic attacks as suggested by some experimental and genetic studies deserves a special attention. Pertinently, the prevalence of PD is more than twice higher in females than in males (Eaton et al. 1994) and synthesis rate of 5-HT is lower in healthy females than in males throughout the cerebral cortex (Nishizawa et al. 1997; Sakai et al. 2006). Furthermore, ATD significantly impaired the recognition of fearful facial expressions in female, but not in male healthy volunteers (Harmer et al. 2003). These findings substantiate the tryptophan-serotonin connection in PD and call for more attention to the vulnerability of 5-HT system in females in the further research on PD and related behaviours.

## Figures and Tables

**Table 1.** Effect of manipulation of 5-HT synthesis rate on the panic responses

**Tryptophan depletion Study**	**Sample description**	**Challenge**	**Study design**	**Hormones**	**Main effects**
Goddard et al. 1994	8 (4 F) PD patients, mean age 42 ± 7 years	None	Double-blind, crossover	Not measured	No exacerbation in panic or anxiety symptoms
Goddard et al. 1995	11 healthy human subjects	yohimbine	Placebo-controlled	Cortisol	Marked increase in feelings of nervousness following the combination test
Kent et al. 1996	5 (2 F) PD patients, mean age 27.2 ± 7.7 years; 7 (2 F) controls, mean age 28 ± 4 years	5% CO2	Double-blind, crossover	Not measured	Significantly increased ventilation in PD patients, but not in controls. No differences on measures of panic or anxiety
Koszycki et al. 1996	40 healthy male volunteers, mean age 24.6 ± 0.9years	CCK-4	Double-blind, parallel-group	ACTH, cortisol, prolactin	No effects on the panicogenic and cardiovascular responses. Significant rise in ACTH/cortisol and prolactin secretion
Klaassen et al. 1998	15 healthy male volunteers, mean age 29 ± 4 years	35% CO2	Double-blind, crossover	Not measured	Significant increase in both anxiety and neurovegetative panic symptoms
Schruers et al. 2000	24 (15 F) PD patients, mean age 40.0 ± 11.5 years	35% CO2	Double-blind, parallel-group	Not measured	Significant increase in both anxiety and panic symptoms
Miller et al. 2000	20 (10 F) PD patients, mean age 38.4 ± 9.9 years 19 (8 F) healthy controls, mean age 29.1 ± 8.4 years	5% CO2	Double-blind, balanced	Cortisol	Greater anxiogenic response and an increased rate of panic attacks in patients. No effects in controls and no significant changes in cortisol levels.
Hood et al. 2006	14 (9 F) healthy subjects, mean age 34.5 (21–60) years	35% CO2	Double-blind, placebo-controlled, crossover	Cortisol, prolactin	No exacerbation of psychological response and no additive effect on endocrine or cardiovascular responses to challenge
Bell et al. 2002	14 (7 F) recovered PD patients, mean age 40.6 (21–65) years	Flumazenil	Double-blind, placebo-controlled, crossover	Not measured	Significantly higher rate of panic attacks and increased cardiovascular responses following ATD and challenge
Davies et al. 2006	27 (12 F) recovered PD (n = 21) or SAD (n = 6) patients, mean age 39.2 ± 12.0 years	Flumazenil; Autobiographical script	Double-blind, crossover	Not measured	Significant increases in acute stress sensitivity in both cardiovascular and psychological domains on 5-HT depletion
Tõru et al. 2006	18 (12 F) recovered PD patients, mean age 34.5 ± 9.3 years	CCK-4	Double-blind, crossover	Not measured	No significant effects of ATD on psychological or cardiovascular responses
**Acute administration of 5-HTP**					
den Boer and Westenberg, 1990	20 female PD patients, mean age 31.3 ± 7.4 years 20 (F 12) healthy controls, mean age 25.0 ± 2.2 years	None	Single-blind, crossover	Cortisol, β-endorphin, melatonin	Relief effect in patients, but not in controls. Substantial and similar increases in hormones
van Vliet et al. 1996	7 (5 F) PD patients, mean age 34.7 ± 12.6 years 7 (4 F) healthy controls, mean age 21.4 ± 3.6 years	None	Double-blind, placebo-controlled	Cortisol	No provocation of panic or anxiety symptoms, but increase in cortisol levels in both groups after 40 mg 5-HTP infusion
Schruers et al. 2002a, b	24 (11 F) PD patients, mean age 40.0 ± 10.7 years 24 (14 F) healthy controls, mean age 29.8 ± 11.7 years	35% CO2	Double-blind, parallel-group	Cortisol	Significant reduction in both panic and anxiety responses in patients, but not in control group. Significant rise in cortisol levels in both groups following 5-HTP administration
Maron et al. 2004b	32 (18 F) healthy subjects, mean age 21.7 ± 2.8 years	CCK-4	Double-blind, parallel-group	Not measured	Significant reduction in panic attacks and cognitive symptoms in females and decrease in somatic panic symptoms in males
